# Monitoring Argon L-Shell Auger Decay Using 250-eV Attosecond X-ray Pulses †

**DOI:** 10.3390/s22197513

**Published:** 2022-10-03

**Authors:** Seunghwoi Han, Kun Zhao, Zenghu Chang

**Affiliations:** 1 CREOL and Department of Physics, University of Central Florida, Orlando, FL 32816, USA; 2 School of Mechanical Engineering, Chonnam National University, Gwangju 61186, Korea; 3 Institute of Physics, Chinese Academy of Sciences, Beijing 100190, China; 4 Songshan Lake Materials Laboratory, Dogguan 523808, China

**Keywords:** attosecond physics, streaking, high harmonic generation, auger decay

## Abstract

Electron correlation describes the interaction between electrons in a multi-electron system. It plays an important role in determining the speed of relaxation of atoms and molecules excited by XUV/X-ray pulses, such as the argon decay rate. Most research on electron correlation has centered on the role of correlation in stationary states. A time-resolved experimental study of electron correlation is a grand challenge due to the required temporal resolution and photon energy. In this research, we investigated Auger decay in argon using 200-attosecond X-ray pulses reaching the carbon K-edge. At such a high photon energy, ionization occurs not only from the outer most levels (*3s* and *3p*), but also from the *2p* core shells. We have measured a lifetime of 4.9 fs of L-shell vacancies of argon in pump–probe experiments with a home-built high-resolution time-of-flight spectrometer.

## 1. Introduction

The transition of an electron from a higher energy level to a core vacancy through various radiative and non-radiative channels is very fast. To release the energy from the transition, the radiative channel emits photons. The non-radiative channel can transfer the energy to another electron, called an Auger electron. The Auger decay is the result of electron-electron interaction, which is a key phenomenon to unveil the effects of electron correlation in complex many body systems.

Observing Auger decay in real time requires a pump–probe technique for initiating the detecting motion of electrons. Time-resolved spectroscopy based on the pump–probe method is a powerful tool for tracking atomic or electronic motion in atoms and molecules with a sub-femtosecond or attosecond pulse laser [1,2,3,4,5,6]. The demonstration of isolated attosecond X-ray pulses in 2001 opened the door to attosecond spectroscopy for sampling photo-electron wave packets [7,8,9,10,11]. Recently, attosecond X-ray pulses reaching the carbon K-edge (282 eV) have been produced by driving high harmonic generation with short-wave infrared lasers [12,13,14,15]. To generate single isolated attosecond pulses, various gating techniques [16,17] and chirp compensation methods [18,19] have been developed. Some of them have been implemented in generating isolated water window X-ray pulses around 50-attosecond pulse width [12,20].

Previous attosecond streaking of Auger electron dynamics in atoms was conducted with extreme ultraviolet (XUV) pulses. The photon energy was less than 100 eV [21,22], which is the cutoff of the high harmonic spectrum obtained with near infrared Ti:sapphire femtosecond pulse lasers. In this paper, we built and used a high-resolution electron time-of-flight (TOF) spectrometer to measure the electron spectra in an X-ray pump–IR probe time-resolved experiment for investigating the individual pathways of the Auger decay with high time evolution. We used 200-attosecond X-ray pulses centered at 250 eV, which photoexcited the L-shell electron of argon atoms and the Auger electron was ejected. The electron binding energies of the L-shell of argon are 248.4 eV for L3 (*2p_3/2_*) and 250.6 eV for L2 (*2p_1/2_*). Then, the probe IR pulse modulated the electron energy. The stable carrier envelope phase (CEP) of the IR pulse led to clear electron energy modulation along with the time delay between the pump and the probe pulses. The home built TOF spectrometer has a spectral resolution of 0.5 eV around 200 eV electron energy. This manuscript is the expanded version of the conference paper [23].

## 2. Materials and Methods

**Figure 1:**
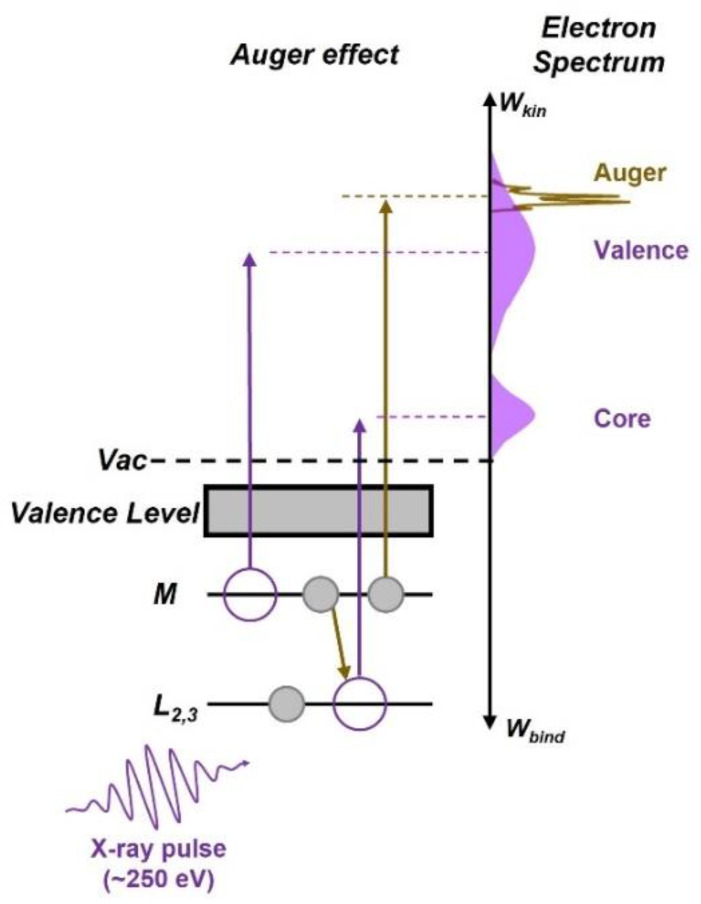
Schematic diagram of argon Auger emission by exposure to an attosecond X-ray pulse.

Figure 1a shows a schematic diagram of the *L_2,3_MM* argon Auger decay process. When an electron in the L-shell of an argon atom is excited by an attosecond X-ray photon with a central energy of 250 eV, an electron in the M-shell fills the vacancy and another electron in the M-shell is released, which is the Auger electron. The measured electrons include that from the Auger decay and the direct photoionization by the X-ray pulse. The electron spectrum of the direct photoionized electron from the M-shell overlaps with that of the targeted Auger signal. Multiple peaks of the *L_2,3_MM* argon Auger spectrum indicate that the photoexcitation emits Auger electrons with different energies by many decay channels [24].

### 2.1. Theoretical Model

The theoretical model is a straightforward extension of the work published in [21,25], which is based on the strong field approximation. This model yields the temporal evolution of hypothetical single-line Auger electron spectra probed by strong few-cycle femtosecond pulse for different decay times. The momentum space wave function of the Auger electron after the passage of both the XUV and the streaking laser pulses can be described by,

(1) $$b\left(p\right)\propto i\int_{t_{0}}^{\infty }exp\left[-i\int_{t^{\prime }}^{\infty }\frac{1}{2}{\left(p-A\left(t^{\prime \prime }\right)\right)}^{2}dt^{\prime \prime }+iW_{kin}t^{\prime }\right]\sqrt{\rho \left(t^{\prime }\right)}\chi \left(p-A\left(t^{\prime }\right)\right)dt’$$

where *W_kin_* is the kinetic energy of the field-free Auger electron, ***A****(t)* is the vector potential of the streaking field, *ρ(t)* is the population of the Auger state, and χ is the initial continuum wave function. The integration has a temporal range of the interaction between the pump–probe pulses and argon atoms. This theory modeled the generation of continuum electrons by an Auger process, which is described by the factors $\sqrt{\rho \left(t^{\prime }\right)}\chi \left(p-A\left(t^{\prime }\right)\right)$. The few-cycle probe pulse is weak, and its influence on the Auger process can be neglected. Therefore, the electron wave function has the same shape as the field-free Auger decay. The strength of the transition to χ is proportional to the Auger decay rate and the *ρ*. We set χ unity in this case because the calculated momentum space wavefunction is insensitive to the exact choice of χ in this model [21].

**Figure 2:**
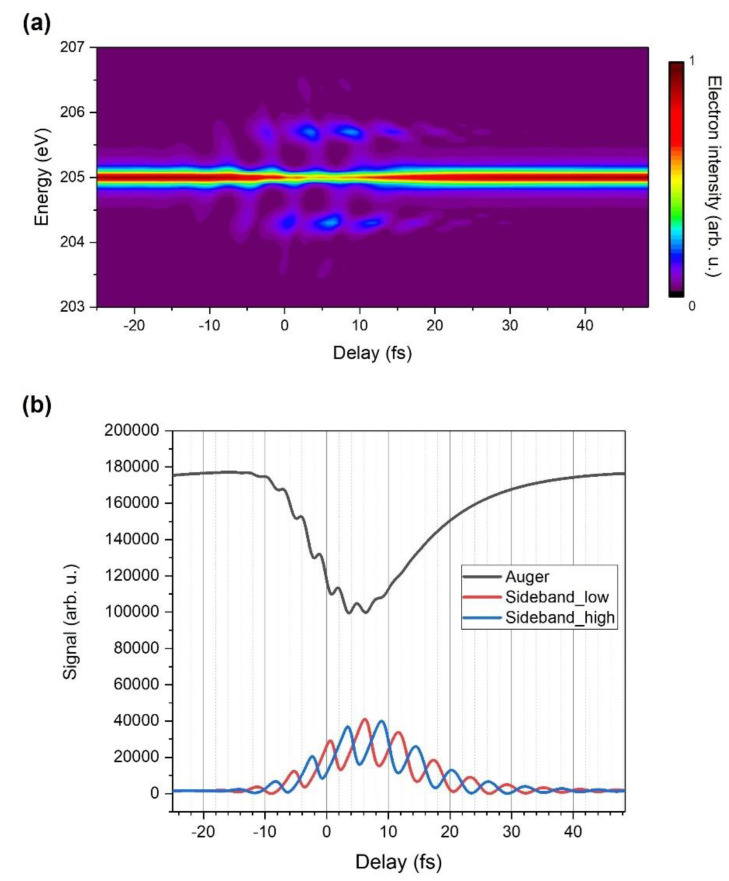
Theoretical calculation [23]. (**a**) Simulated streaking spectrogram of an argon Auger decay channel. (**b**) The envelopes of the calculated Auger and sideband lines.

Figure 2a shows the calculated streaking spectrogram of a single Auger channel resulting from a hypothetical single-line atomic core hole delay with a 5-fs decay time constant in the presence of a two-cycle 1.7-µm laser field with zero CEP. The optical period of the field is 5.67 fs. When the Auger lifetime is comparable to the laser cycle, which is relevant to this experiment, the energy distribution of the Auger electron is broadened and broken up into sidebands spaced by the photon energy of the IR laser field (~0.7 eV). Figure 2b indicates that the envelopes of the Auger and sideband lines are the results of the convolution of the Auger decay rate and IR pulse envelope. Two side bands are out of phase due to the momentum of the electron obtained from the oscillating IR field.

The theoretical model used for the calculations is an extension of the single-line case. We assumed that the total wave function of the outgoing electron is a superposition of Auger electron wave functions corresponding to all decay channels,

(2) $$b_{total}\left(p\right)=\overset{N}{\underset{i=Auger\;peaks}{\sum }}a_{i}b_{i}\left(p\right)$$

where *a_i_* is the coefficient of the Auger electron related to the relative intensity in Table 1.

**Figure 3:**
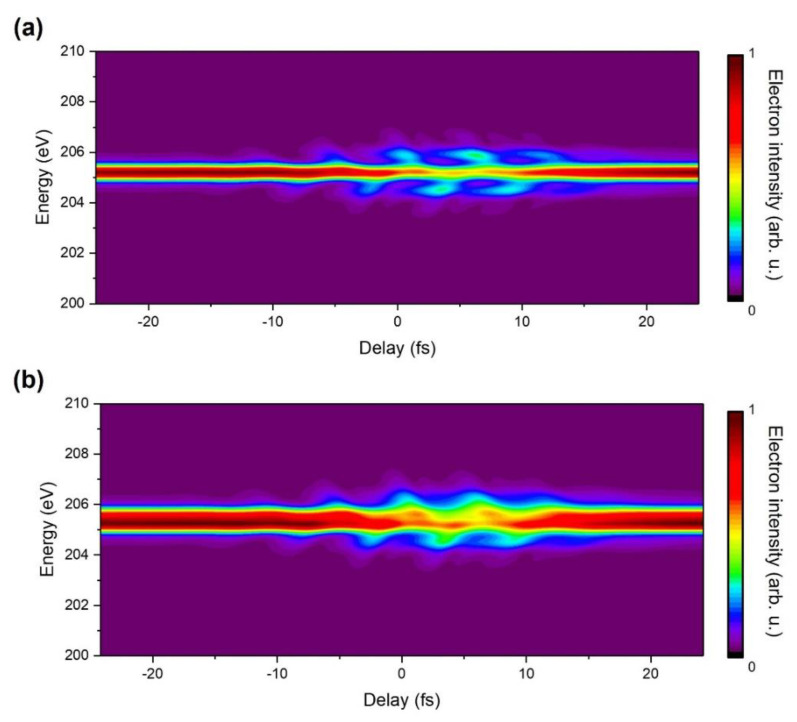
(**a**) Calculated spectrogram of the argon Auger emission of 205.21 eV with the convolution effect which is related to the spectral resolution of the experimental setup. (**b**) The calculated spectrogram of two close argon Auger emissions of 205.21 eV and 205.62 eV.

Figure 3a shows the calculated argon Auger electron spectrogram convoluted with the spectral resolution of the TOF. The measured spectral resolution is about 0.5 eV at 205 eV when the retarding potential was set to 190 V. The kinetic energy of the argon Auger electron for one of the interest channels is 205.21 eV. The Auger peak and sidebands are broadened along the energy axis due to the convolution effect compared to Figure 2a. Figure 3b shows the calculated two closely spaced Auger peaks, 205.21 eV and 205.62 eV, taking into account the differences in their intensity coefficients. The merged spectra show an oscillation at the IR laser frequency, although it is hard to resolve the two peaks due to the limited energy resolution of the experimental setup.

### 2.2. Experimental Setup

**Figure 4:**
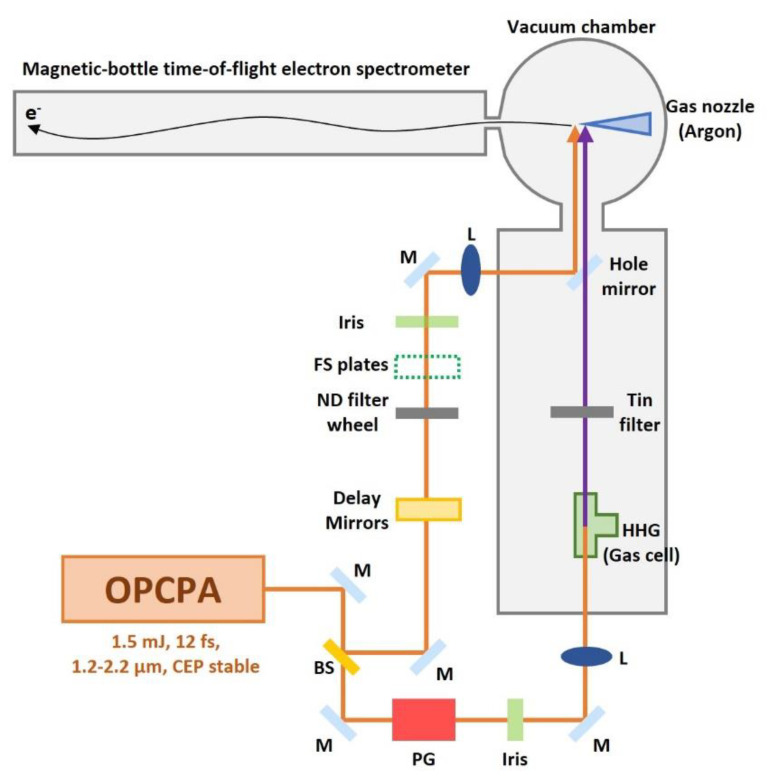
Schematic illustration for argon Auger decay measurement using isolated X-ray attosecond pulse and a long magnetic-bottle time-of-flight electron spectrometer for higher spectral resolution. M: mirror, BS: beam splitter, L: focusing lens, FS plates: fused silica plates, PG: polarization gating optics. The inset picture shows the 7-m-long magnetic-bottle time-of-flight electron spectrometer.

Figure 4 illustrates a schematic diagram of the experimental setup. X-ray pulses were generated from high harmonic generation driven by a 1-kHz, CEP stabilized, optical parametric chirped pulse amplifier (OPCPA) system with an output of a 12-fs (two-cycle) pulse near 1.7 µm [26]. The pump laser for the OPCPA is a home-built Ti:sapphire chirped pulse amplifier (CPA) system with synchronized output of 2.2 mJ, 30 fs pulses and 18 mJ, 5 ps pulses. The 30 fs pulses are focused into a neon-filled hollow-core fiber for bandwidth broadening by white light generation. The broadband white light pulses are compressed to 7 fs by chirped mirrors then focused into a BIBO crystal for intra-pulse difference-frequency mixing [27]. This yields a broadband IR seed pulse with passive CEP stability. These IR seed pulses are stretched to 4.4 ps using an acousto-optic programmable dispersive filter [28] and amplified by BIBO-based OPCPA stages, which use the synchronized output of the 18 mJ, 5 ps pulses from the Ti:sapphire CPA system as pump pulses. The amplified IR pulses are compressed to 12 fs using a material dispersion of bulk fused silica. The output IR pulse of the OPCPA has 1.5 mJ pulse energy.

The IR beam is split by a beam splitter into an X-ray generation arm (90%) and an IR streaking arm (10%) [12]. In the X-ray generation arm, polarization gating has been adopted to generate broadband isolated attosecond pulses. The polarization gating optics convert a linearly polarized pulse into one within which the field polarization changes from circular to linear and back to circular. The OPCPA output after the polarization gating is loosely focused (f = 450 mm) into a 1.5 mm-long neon gas cell to generate <200 attosecond isolated soft X-ray pulses centered at 200 eV [12]. Backing pressure of the neon gas cell is 1 bar. A tin (Sn) filter of 400 nm thickness is inserted to block the residual IR beam and to compensate for the chirp of the XUV pulses. The XUV pulses are retrieved by the phase retrieval by omega oscillation filtering (PROOF) [12]. The soft X-ray beam is focused through a hole-drilled mirror (hole diameter = 3.5 mm) by a toroidal mirror. In the streaking arm, there is a variable time delay setup. Fused silica plates compensate the chirp of the IR pulses. The hole-drilled mirror combines the X-ray and the IR streaking pulses. The two pulses have a collinear beam path and are focused onto the argon gas from a nozzle to generate Auger electrons and photoelectrons, which are collected by a 7-m-long magnetic bottle electron time-of-flight spectrometer [29]. The interference fringe from a continuous wave green laser (center wavelength = 532 nm) co-propagating with the IR beam through both arms are used to feedback control a PZT that controls and stabilizes the time delay between the X-ray and the IR streaking pulses [12].

**Figure 5:**
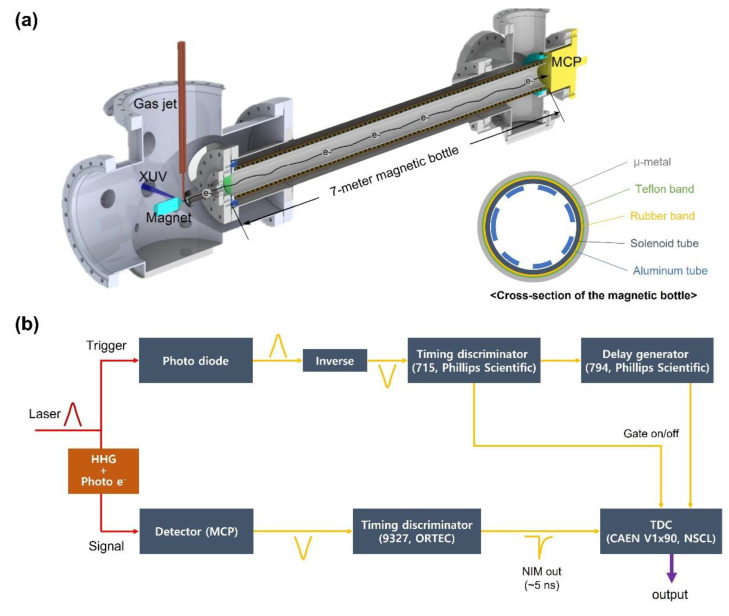
High resolution electron time-of-flight spectrometer. (**a**) Diagram of the magnetic bottle electron spectrometer with a seven-meter-long flight tube [29]. The XUV beam is focused between a magnet and an aperture of the spectrometer. A gas jet is placed on top of the focusing spot of the XUV beam. Generated photoelectrons enter the aperture of the spectrometer and propagate through the seven-meter-long tube then detected by a MCP detector. (**b**) Data process for measuring the photoelectron energy.

Figure 5a indicates a schematic drawing of the spectrometer. The focused XUV beam ionizes the core electron of argon molecules injected from a gas jet. A strong permanent magnet with a conical pole piece produces a diverging solid magnetic field. The strong magnetic field drives the photoelectrons to collimate. A portion of the collimated photoelectrons enter the aperture of the spectrometer, and the constant magnetic field of the flight tube guides the photoelectrons along the seven-meter-long TOF spectrometer. A solenoid coil covered by an inner aluminum tube produces a constant magnetic field inside the tube. The whole tube is covered by a μ-metal tube to shield the earth’s magnetic field. To protect the solenoid coil, multiple rubber bands and Teflon bands are placed between the solenoid tube and the μ-metal tube.

The temporal resolution of the electron TOF spectrometer is a function of the tube length and the parallelization of the photoelectrons. Different emission angles of electrons make different flight times along the TOF tube. The longer flight tube has a better temporal resolution. The final system resolution is a result of convolution with the temporal resolution of the data acquisition system.

To enhance the spectral resolution over the 200 eV range, we extended the magnetic bottle length from 3-m to 7-m and applied a retarding potential to slow down electrons. The longer the magnetic bottle, the better the temporal resolution due to the increased electron flight time in the spectrometer. The retarding potential can increase the flight time of the charged particles significantly. In the magnetic bottle entrance, two metal meshes of 90% transmission separated by 5 mm are placed. The first mesh is grounded, and a retarding potential is applied to the second one, which is electrically connected to the rest of the magnetic tube.

Figure 5b shows the data acquisition system of the electron TOF spectrometer. When the MCP detects the photoelectron signal, a fast voltage signal is produced. Then, a timing discriminator (constant fraction discriminator) determines the arrival time of the signal. A time-to-digital converter (TDC) digitizes the analog arriving time, and the time data are stored in the computer. To judge the arrival time, a photodiode before the chamber detects the portion of the XUV pulse and gives a reference time. The measured resolution of the spectrometer is 0.5 eV at 205 eV.

## 3. Results and Discussion

**Figure 6:**
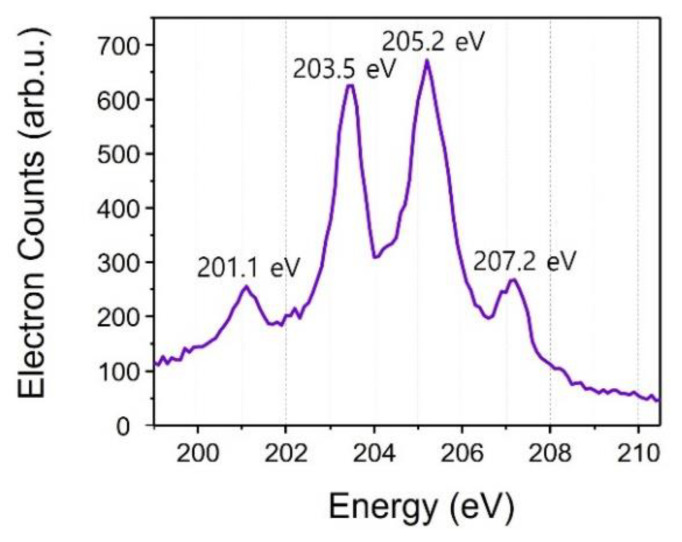
Argon Auger photoelectron spectrum without the IR pulse.

The violet energy spectrum in Figure 6 is the Auger electron spectrum by X-ray pulses only. The spectrum shows four argon Auger peaks clearly. Applying a retarding potential of 190-V enhances the spectral resolution of the spectrometer. Two strong peaks (203.5 eV and 205.2 eV) consist of two closely spaced Auger peaks each. Two adjacent Auger electron peaks show similar structure as the calculated one, as shown in Figure 3b.

**Figure 7:**
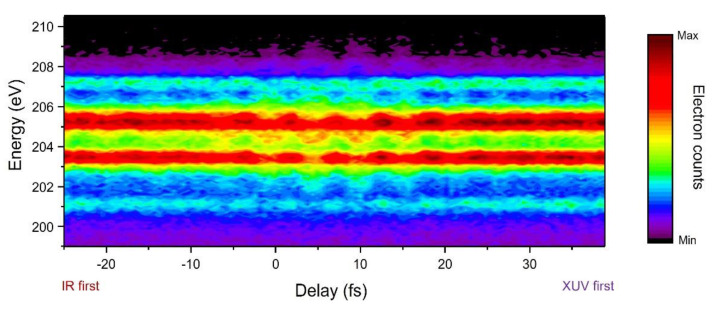
Attosecond streaking spectrogram.

Figure 7 shows the evolution of electron spectra following core excitation. The spectra were recorded after exposure of argon atoms to a 200-attosecond soft X-ray pulse and a femtosecond IR pulse at different delays between them. A negative delay corresponds to an earlier IR pulse arrival. The plot shows the evolution of the *L_2,3_M_2,3_M_2,3_* Auger lines with delay. The IR laser peak intensity was evaluated as $I_{p}=2\times 10^{11}\;W/cm^{2}$. The IR laser field spreads the kinetic energy of the Auger electrons and induces clear sideband structures. The evolution of the first-order sidebands of the two strong Auger peaks (203.5 eV and 205.2 eV) shows strong modulation along the delay. The Auger peak of 207.2 eV also oscillates with the same frequency and phase as the two strong peak’s oscillation. It means that the Auger electrons from these channels have the same initial phase. The peak at 201.1 eV is too weak to see any clear structures.

**Figure 8:**
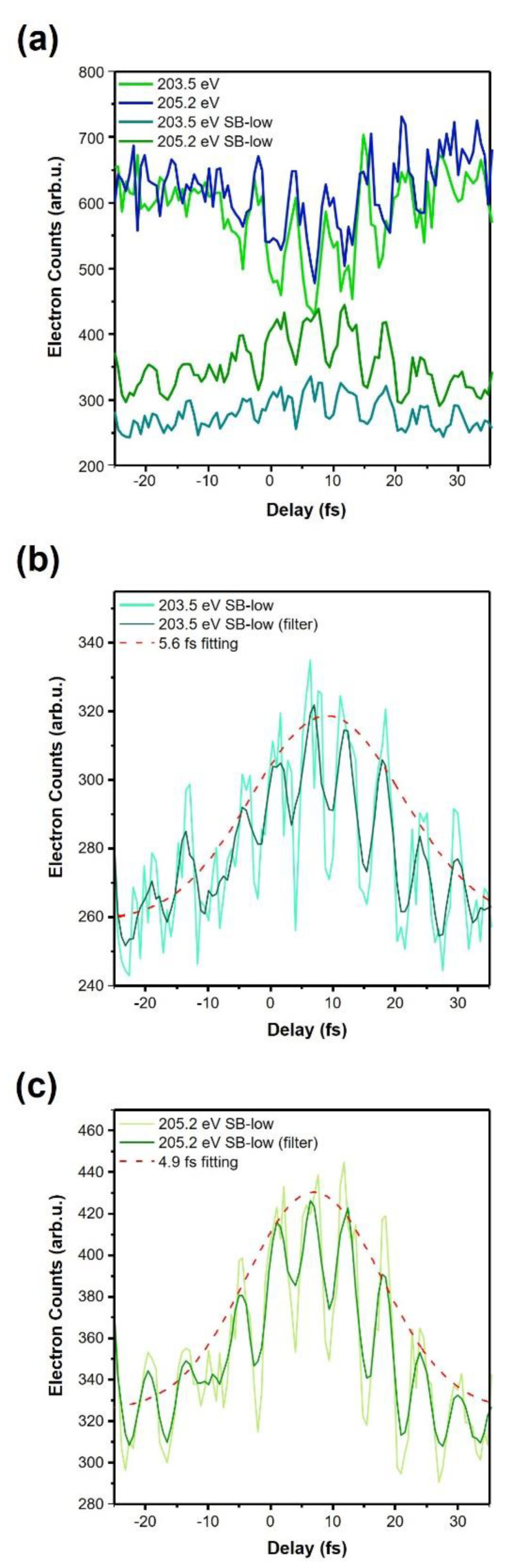
Probing the temporal evolution of argon Auger electron emission. (**a**) Measured electron counts of two strong Auger peaks and corresponding sidebands along the delay. (**b**) The extracted argon Auger decay time of 203.5 eV Auger peak from the sideband of 202.8 eV. (**c**) The extracted argon Auger decay time of 205.2 eV Auger peak from the sideband of 204.5 eV.

Figure 8a shows the measured electron counts of two strong Auger peaks and corresponding sidebands along the delay. The sideband at 202.8 eV (203.5 eV SB-low) is the first-low-order of the Auger peaks at 203.5 eV. The sideband at 204.5 eV (205.2 eV SB-low) is the first-low-order of the Auger peaks at 205.2 eV. According to the theoretical results, higher and lower order sidebands are out of phase. Two neighboring Auger peaks may affect the oscillation of Auger peaks along the delay because one Auger peak’s sideband signal may merge with the other Auger peak. Figure 8b shows the extracted argon Auger decay time (*τ_h_*) of the 203.5 eV peak (*L_3_M_2,3_M_2,3_*(*^1^D_2_*)) is 5.6 fs. The measured decay time of the 205.2 eV peak (*L_3_M_2,3_M_2,3_*(*^3^P_0,1,2_*)) is 4.9 fs (Figure 8c). The sideband profiles are fitted by the convolution of the Auger decay rates and the IR pulse envelope functions. The values of the delay axis are changed for a fitting. These values correspond to linewidths (*Γ =h/τ_h_*) of 117 meV and 134 meV. The extracted linewidths are in good agreement with the spectroscopic measurement, which reported that the measured inherent lifetime widths from the spectroscopy of the Ar *2p_1/2_* and Ar *2p_3/2_* are both 118 meV [30]. There is no difference in the inherent lifetime width for the spin-orbit split components of Ar *2p* ionized states. The extracted lifetimes of the multiple Auger signals with similar envelopes also agree with [30].

The modulation of the Auger peaks comes from the combined effects of multiple Auger peaks that are very close in the spectral domain. From the measurement, we can find out that the four Auger electrons have almost the same lifetime and phase. Another Auger peak with 207.2 eV shows an oscillation with the same phase of these strong Auger peaks and is similar to the theoretical results for the single Auger peak.

The XUV pulses react with electrons in the argon atom. The ionization energy of the outermost argon electron (*3p*) is 15.76 eV. The generated photoelectrons from the outermost shell are also dissipated in momentum space by the probe’s few-cycle femtosecond pulses. These signals can be measured with the measured attosecond streaking spectrogram of the Auger electron when the retarding potential is zero. However, when the retarding potential of 190 V was applied to increase the resolution of the TOF spectrometer, the photoelectron streaking signal was not measured. Therefore, photoelectrons from argon *3s* or *3p* do not have a significant effect on this modulation of Auger peaks. 

## 4. Conclusions

We investigated the argon L-shell Auger decay with attosecond streaking at ~200 eV electron energy. It demonstrated that the flux of the isolated attosecond X-rays generated by the 1.7 μm lasers is sufficient for studying the dynamics of electrons in deep inner shells with over 250-eV binding energy. To the best of our knowledge, this is the first time that streaking of Auger electrons has been measured by exciting atoms with over 100 eV attosecond photons, which paves the way to study interatomic Coulomb decay in organic and other molecules.

## Figures and Tables

**Table 1:** Energies and assignment of the argon Auger lines in this experiment [24].

Kinetic Energy of Argon Auger Peak (eV)	Assignments	Relative Intensity (arb. u.)
201.09	*L_3_M_2,3_M_2,3_(^1^S_0_)*	100
203.23	*L_2_M_2,3_M_2,3_(^1^S_0_)*	55
203.47	*L_3_M_2,3_M_2,3_(^1^D_2_)*	270
205.21	*L_3_M_2,3_M_2,3_(^3^P_0,1,2_)*	272
205.62	*L_2_M_2,3_M_2,3_(^1^D_2_)*	183
207.23	*L_2_M_2,3_M_2,3_(^3^P_0,1,2_)*	139

## Data Availability

Not applicable.
